# Citizen science substantiates jellyfish occurrence in the Mediterranean Sea

**DOI:** 10.1038/s41598-025-05789-1

**Published:** 2025-07-01

**Authors:** Serena Zampardi, Priscilla Licandro, Giacomo Milisenda, Danilo Scannella, Stefano Piraino, Ferdinando Boero

**Affiliations:** 1 Institute for Environmental Protection and Research (ISPRA), BIO-CIT, Palermo, 90149 Italy; 2 Department of Integrative Marine Ecology, Stazione Zoologica Anton Dohrn, Villa Comunale, Naples, 80121 Italy; 3 Department of Integrative Marine Ecology, Sicily Marine Centre, Stazione Zoologica Anton Dohrn, Palermo, 90100 Italy; 4 Dipartimento di Scienze e Tecnologie Biologiche e Ambientali (DiSTeBA), Università del Salento, Lecce, 73100 Italy; 5 National Biodiversity Future Center (NBFC), Palermo, 90100 Italy; 6 Consorzio Nazionale Interuniversitario per le Scienze del Mare (CoNISMa), Rome, 00196 Italy; 7 Dipartimento di Biologia, University of Naples Federico II, Naples, 80134 Italy; 8 Consiglio Nazionale delle Ricerche, Istituto per lo Studio degli Impatti Antropici e Sostenibilità in Ambiente Marino (CNR-IAS), Genoa, 16149 Italy

**Keywords:** Gelatinous organisms, Non-indigenous species (NIS), Geographical distribution, Bloom, Hot-spot areas, Ecology, Zoology

## Abstract

In recent decades, gelatinous plankton blooms have attracted media attention due to their damaging effects on fisheries, aquaculture, tourism, human health and power/desalination plants. The scientific community often failed to recognize the rise of jellyfish, due to difficulties in monitoring their appearances and disappearances. In Italian waters, a citizen science approach was employed to monitor the presence and distribution of gelatinous zooplankton over wide spatio-temporal scales. From 2009 to March 2016, citizens identified 27 jellyfish taxa and documented their geographical distribution and seasonal occurrence across the Italian Seas. The main blooming species was *Pelagia noctiluca* (30% of total sightings), mainly distributed in the western Mediterranean, followed by *Rhizostoma pulmo* (28% of total sightings), showing a more widespread distribution. *Mnemiopsis leidyi*, the main Non-Indigenous Species (NIS), initially reported in the western Mediterranean, since 2016 expanded its distribution to the east. Jellyfish blooms were recorded year-round, mainly between late spring and autumn. Jellyfish hotspots were identified in the northern and southern Tyrrhenian, Ligurian Sea, and northern Adriatic regions. Citizen Science enables the detection of phenomena that can be studied more in-depth by professional scientists, leading to the discovery of previously unknown species and a better understanding of the occurrence of bloom events.

**Supplementary Information:**

The online version contains supplementary material available at 10.1038/s41598-025-05789-1.

## Introduction

Since the early 1980s, increasing proliferations (i.e. blooms) of jellyfish (i.e. gelatinous zooplankton comprising carnivorous and herbivorous invertebrates belonging to the phyla Cnidaria, Ctenophora and Cordata) have been reported from the Mediterranean Sea, where historical data on jellyfish occurrence are available^1–3^. Jellyfish blooms are now more frequent than in the past and regularly occur the whole year-round, including “cold” wintertime, as opposed to what was observed in the previous 60–100 years^4,5^.

Mills (2001)^6^ was the first to suggest that changes in gelatinous plankton abundance evidenced a global trend. Regional studies later confirmed this tendency^7–11^. A global analysis further showed that jellyfish swarms reported in fish catches have increased of about 42% within large marine ecosystems worldwide since the 1950s^12^.

Some members of the scientific community questioned a true global jellyfish rise, invoking phases of periodic fluctuations and/or regional phenomena^13,14^, whose “irregularities” can significantly affect ecosystem functioning^15^. Due to their impacts, jellyfish bloom events have rapidly gained media attention, since they have been increasingly reported as affecting economic activities, including fisheries, tourism, industry and aquaculture worldwide^10,16,17^.

Stakeholders and policy makers^18^ are increasingly called to evaluate the risk associated with jellyfish blooms and to implement measures that can possibly reduce their detrimental impacts on the marine environments under their jurisdiction. However, apart from a few exceptions, records on jellyfish abundance and diversity are scarce and still need to be more comprehensive to allow any prediction of where and when bloom events will occur. Past and current plankton monitoring programs typically do not cover jellyfish due to sampling limitations and lack of taxonomic expertise^19^, while remote sensing techniques are still inadequate to detect jellyfish. Citizen Science (CS), gathering jellyfish sightings by scientists, amateurs and the public, has been successful in monitoring jellyfish at a broad spatial scale, identifying regions where jellyfish-related disturbances are perceived^20,21^. CS has been already successful in pinpointing changes in seasonal cycles and the geographical distribution of terrestrial animals and plants^22–24^ and is increasingly employed in monitoring seawater quality and living marine resources, such as intertidal organisms, invertebrates, fish, seabirds and mammals^25^. Here we use eight years of CS data (2009–2016) to test the effectiveness of Citizen Science in monitoring jellyfish abundance and diversity along 8,500 km of Italian coasts. As regional monitoring programs do not provide jellyfish data series at high taxonomic resolution, our aim is to verify whether distributional patterns reported by CS for sighted jellyfish are recurrent over time (i.e. season) and space and consistent with the few observations available.

CS has already proven successful in providing reliable records of key jellyfish species, leading to mapping their distribution over time and identifying sub-regions primarily affected by bloom events in different periods of the year^10,20,26–34^.

## Materials and methods

**Fig. 1:**
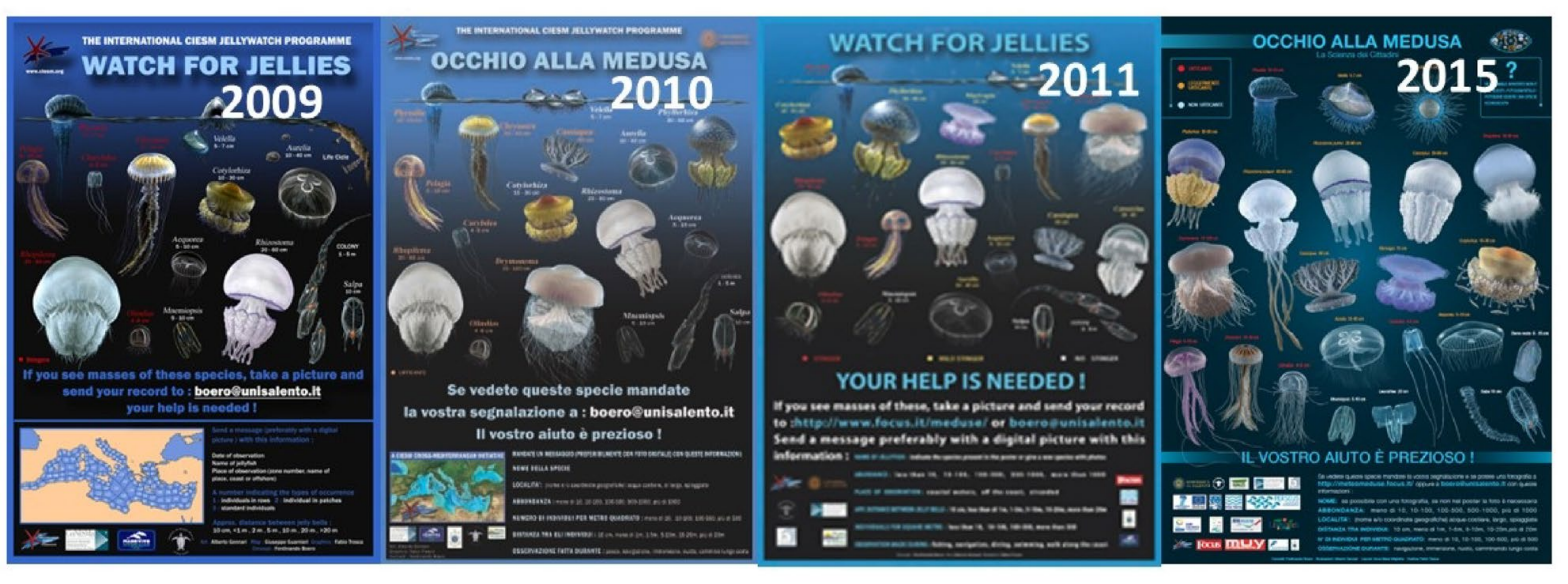
Posters of the main gelatinous species of the Mediterranean Sea realized from 2009 to 2015 by the Prof. Ferdinando Boero. Artwork: Alberto Gennari, Graphic work: Fabio Tresca.

The CIESM Jellywatch Program was launched in 2008 with a pilot project in Italy (“Occhio alla Medusa: Spot the Jellyfish”). The first poster illustrating the main gelatinous species of the Mediterranean Sea, including also the most conspicuous gelatinous alien species, was produced and distributed in 2009, with regular updates printed in the following years (Fig. 1). Regular press releases and TV coverages boosted the campaign’s visibility, engaging an increasing number of citizens. In 2010, the most popular Italian science magazine, FOCUS, supported the campaign, printing the jellyfish poster as centrefold in the summer issue, creating a dedicated webpage on its portal, and releasing a smartphone app to upload records. The initial 2009 poster illustrated 13 jellyfish species, expanding to 16 in 2010 and 18 in 2011. In 2015, the number of conspicuous taxa of gelatinous zooplankton as depicted in the poster, rose to 23. The FOCUS web page dedicated to the campaign featured descriptions and numerous images of each species listed on the poster.

Sightings data were checked and promptly released on the FOCUS web page within daily jellyfish sightings maps that triggered a wave of popularity on the initiative. However, in 2015, the collaboration with FOCUS ended, leading to the closure of both website and the app that collected the data.

**Fig. 2:**
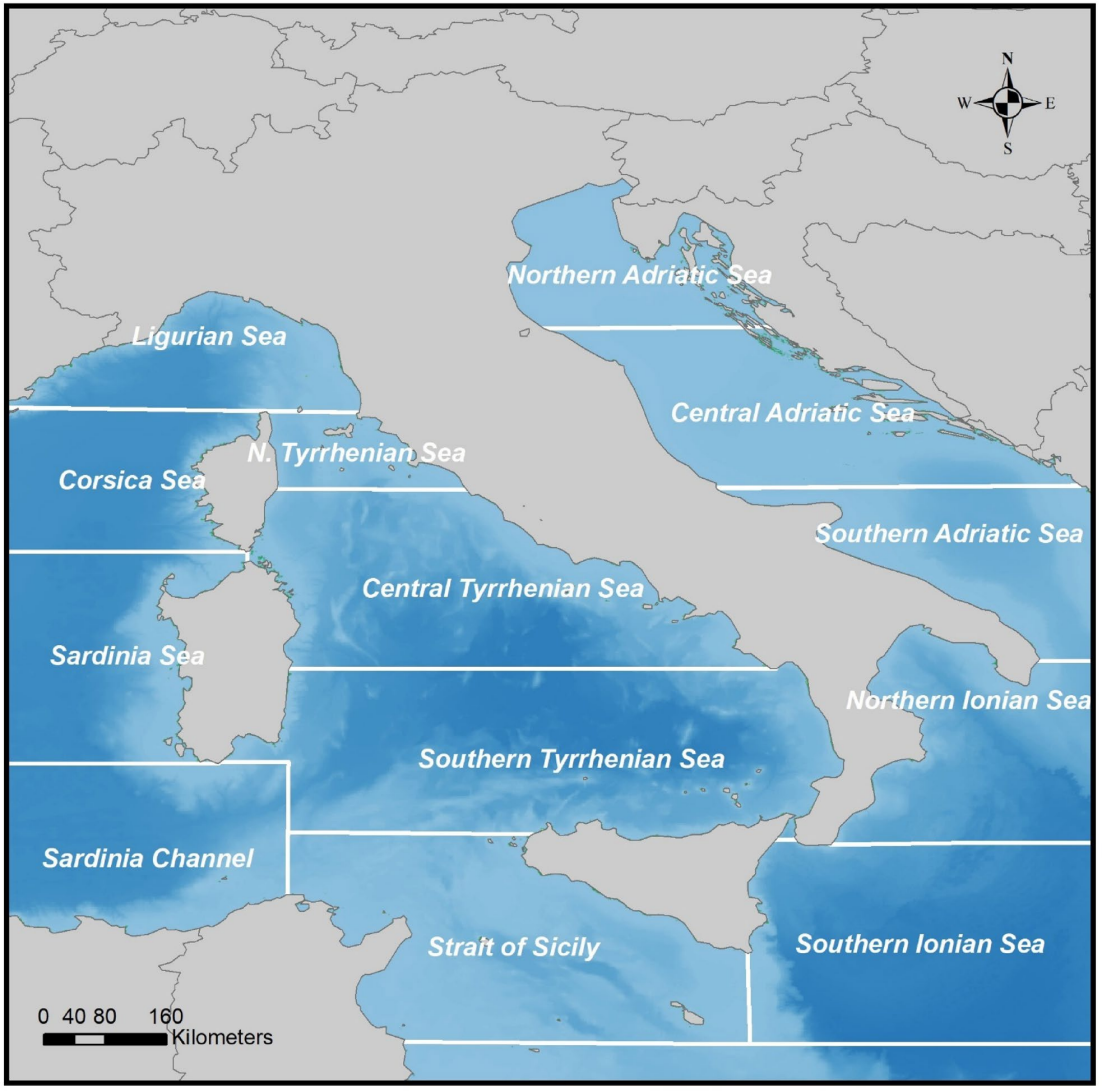
Map of the Mediterranean sectors bordering the Italian coastline. Map generated with ArcMap 10.8.2 (Esri, https://www.esri.com/en-us/arcgis/products/arcmap/overview).

During the period 2009–2016, citizens collected more than 19,000 records, supervising 8,500 km of Italian coastline, belonging to 13 Mediterranean sectors (Northern, Central and Southern Tyrrhenian Sea, Ligurian Sea, Strait of Sicily, Sardinian Sea, Sardinia Channel, Corsica Sea, Northern, Central and Southern Adriatic Sea and Northern and Southern Ionian Sea), 815 Municipalities and 63 Districts (Fig. 2). Records included details such as date and location of the observation, the sighted genus or species, and its ranked abundance according to five predefined categories (number of individuals per square meter: <10, 10–100, 100–500, 500 − 100, > 1000). Additional information on the observation method and a photograph were also recorded.

Records of more than ten individuals per square meter were classified as blooms, defined here as the aggregation of many individuals, usually due to wind or current patterns^35^. Records of some species were considered valid in the absence of pictures, whenever they were confirmed by pictures received from the same area on the same day or week. Records of unusual species were only accepted if documented by pictures. Unreliable records were discarded. The reports of new species were provided by the University of Trieste and the University of Croatia, which conducted morphological and genetic analyses to confirm their taxonomic identification.

All checked jellyfish bloom sightings (*N* = 6667), validated by experts, were assembled into a georeferenced database. To analyse the spatial distribution of blooms, we applied a Kernel Density Estimation (KDE) approach using the Point Density tool in ArcGIS software (ArcMap 10.1, Esri, www.esri.com)^36^ following Silverman (1986)^37^. KDE estimates the density of sightings around each raster cell by defining a neighbourhood with a fixed search radius (1 km in our case), summing the number of sightings within this neighbourhood.

Based on the KDE outputs, we identified jellyfish hotspots as areas with high estimated densities. Specifically, for each species, hotspot cells were defined as those with KDE values equal to or exceeding 60% of the species-specific maximum density.

To evaluate spatial co-occurrence among species, we calculated the Jellyfish Hotspot Overlap Index (JHOI) which quantifies the number of species sharing a hotspot in each raster cell. The JHOI was computed using the following formula:

1 $$\:JHOI=\frac{1}{k}\sum\:_{s=1}^{k}{\delta\:}_{is}$$

where *K* is the total number of species (*s*) and δ_*is*_ = 1 if the KDE value in cell *i* for species *s* is ≥ of the species’ maximum KDE value, or δ = 0 otherwise.

This raster-based index ranges from 0 (no spatial overlap of species-specific hotspots) to 7 (complete overlap of all species considered: *Aurelia spp* (Lamarck, 1816), *Pelagia noctiluca* (Forsskal, 1775), *Rhizostoma pulmo* (Macri, 1778), *Carybdea marsupialis* (Linnaeus, 1758), *Cotylorhiza tuberculata* (Macri, 1778), *Velella velella* (Linnaeus, 1758), *Mnemiopsis leidyi* (Agassiz, 1865)) (complete interspecific spatial overlap). Maps of $\:JHOI$ were produced in ArcGIS to visualize patterns of interspecific hotspot overlap.

The resulting index quantifies the extent to which jellyfish species co-occur spatially, providing insight into potential ecological interactions and competition among species. The Kruskal-Wallis test, a non-parametric analysis, was applied to assess statistically significant differences among Italian marine sectors considered in this study. Notched boxplots were produced for each marine sectors for the graphical representation of the distributions. The sectors were displayed following a North-South latitudinal gradient: Northern Adriatic (NA), Ligurian Sea (LS), Corsica Sea (CS), Northern Tyrrhenian (NT), Central Adriatic (CA), Central Tyrrhenian (CT), Southern Adriatic (SA), Sardinian Sea (SS), Southern Tyrrhenian (ST), Northern Ionian (NI), Sardinian Channel (SC), Strait of Sicily (SoS), Southern Ionian (SI).

## Results

### Citizen science sightings

**Fig. 3:**
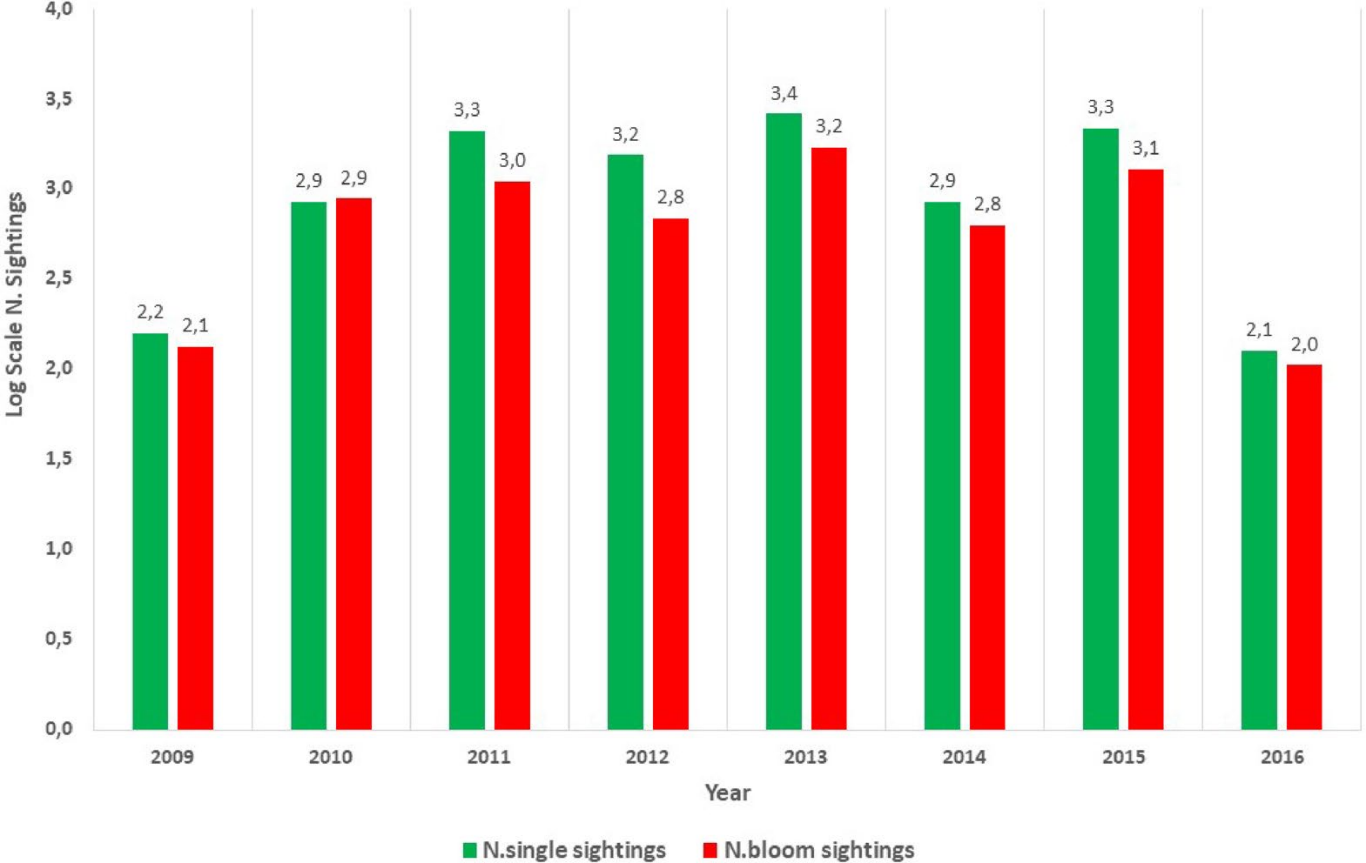
Total number (log scale) of sightings and bloom events (> 10 ind/m^2^) recorded from 2009 to 2016.

Between 2009 and 2016, the CIESM “Jellywatch” Citizen Science Program recorded more than 19,000 sightings, including about 7,000 bloom events across 27 taxa, comprising Hydrozoa, Scyphozoa, Cubozoa, Ctenophora, Tunicata and a few unidentified species^38^ (Fig. 3, Tab.S1). Most sightings occurred from June to September, when beaches are generally more visited by people (Fig.S1-S5). The availability of a dedicated app and website corresponded to an increase in the number of reports (Fig. 3), contributing to an expanded diversity of species recorded between 2010 and 2015.

The highest number of sightings was documented in 2013. In contrast, in 2009 and 2016, when the app and the web page were not available, a lower number of sightings were recorded, possibly due to the switch to email-only reporting.

**Fig. 4:**
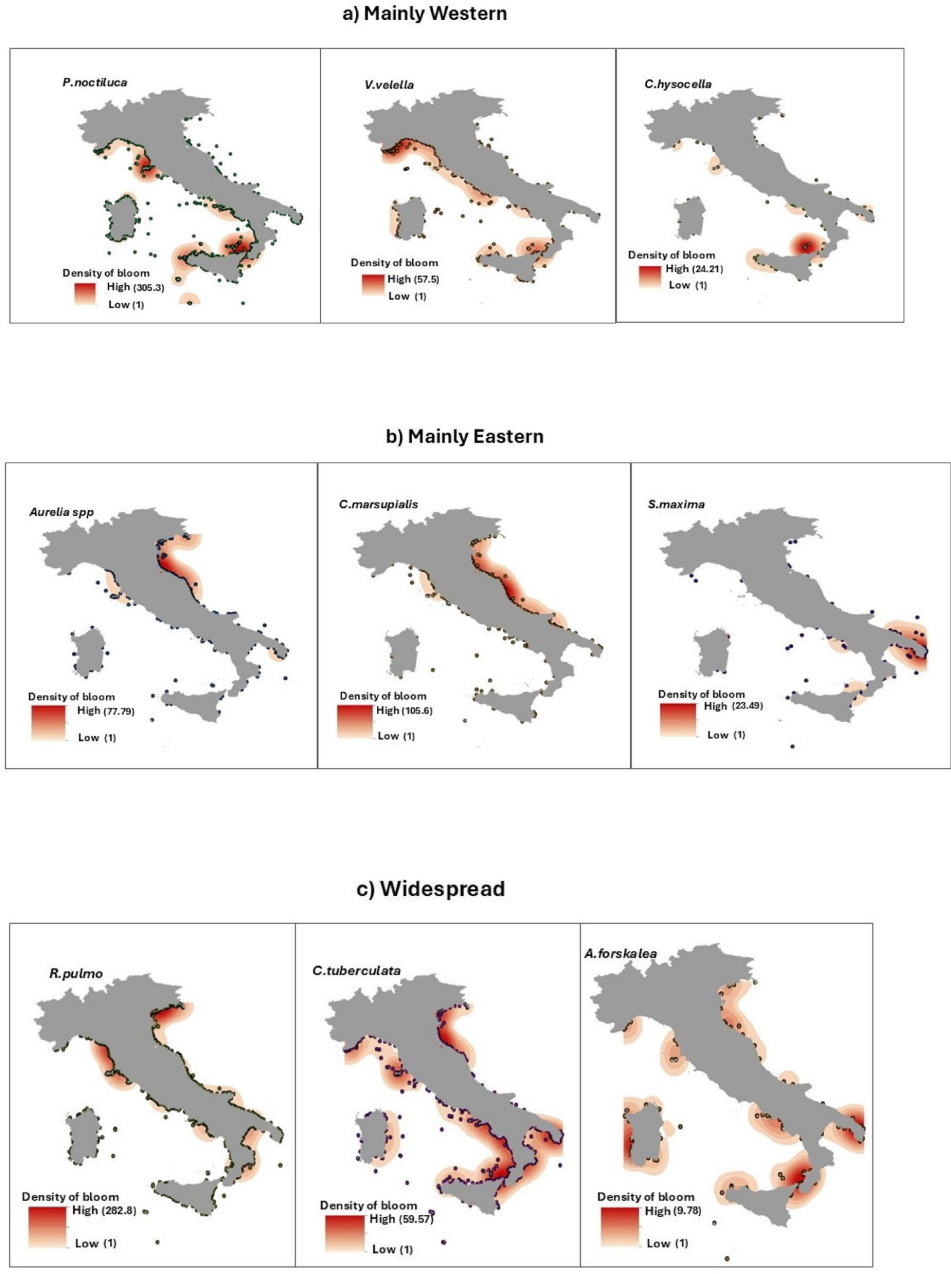

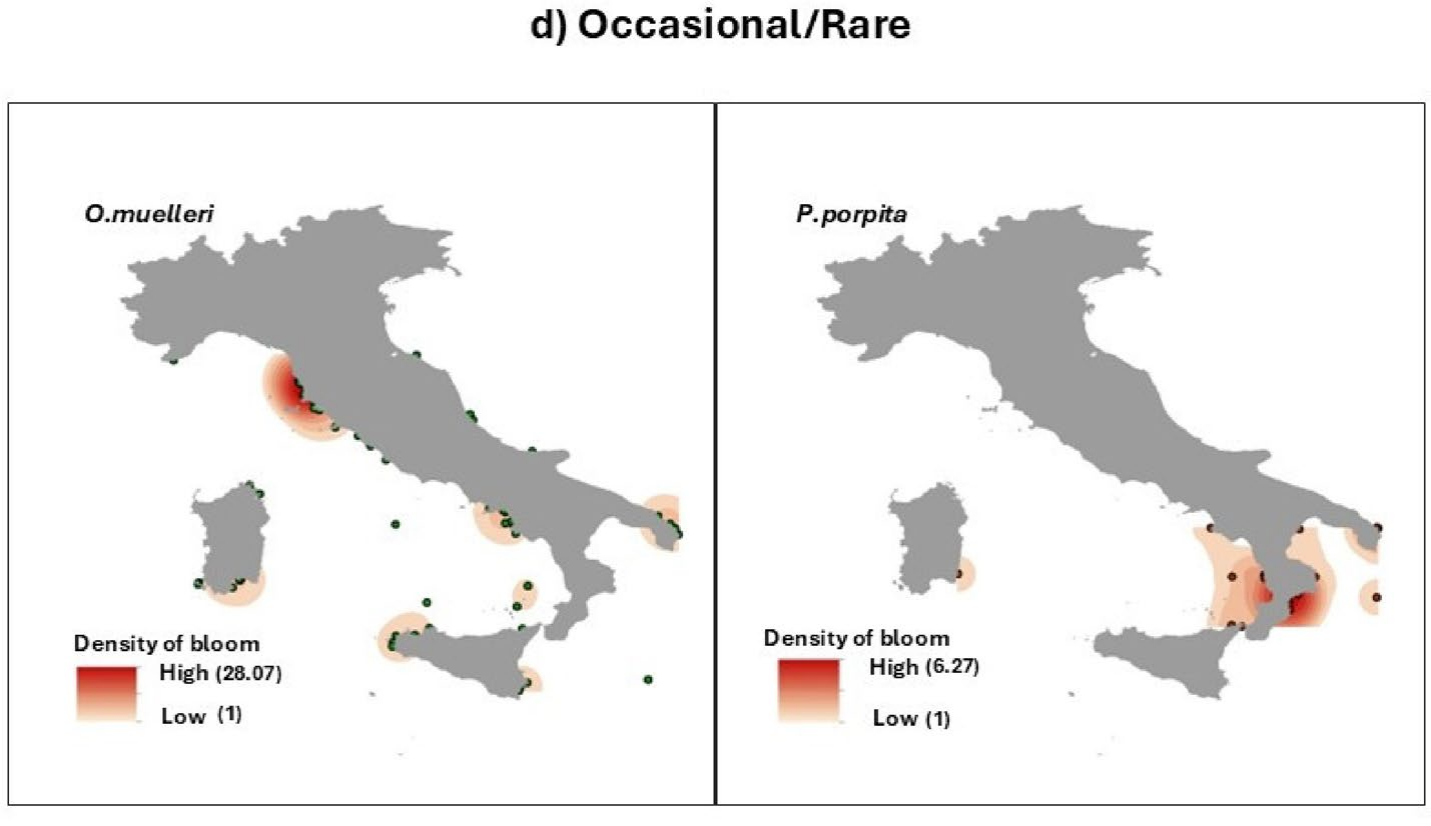
Maps of distribution of most frequent indigenous species (IS) from 2009 to 2016 categorized as predominantly western (**a**), eastern (**b**), widespread (**c**) or occasional/rare (**d**). Map generated with ArcMap 10.8.2 (Esri, https://www.esri.com/en-us/arcgis/products/arcmap/overview).

**Fig. 5:**
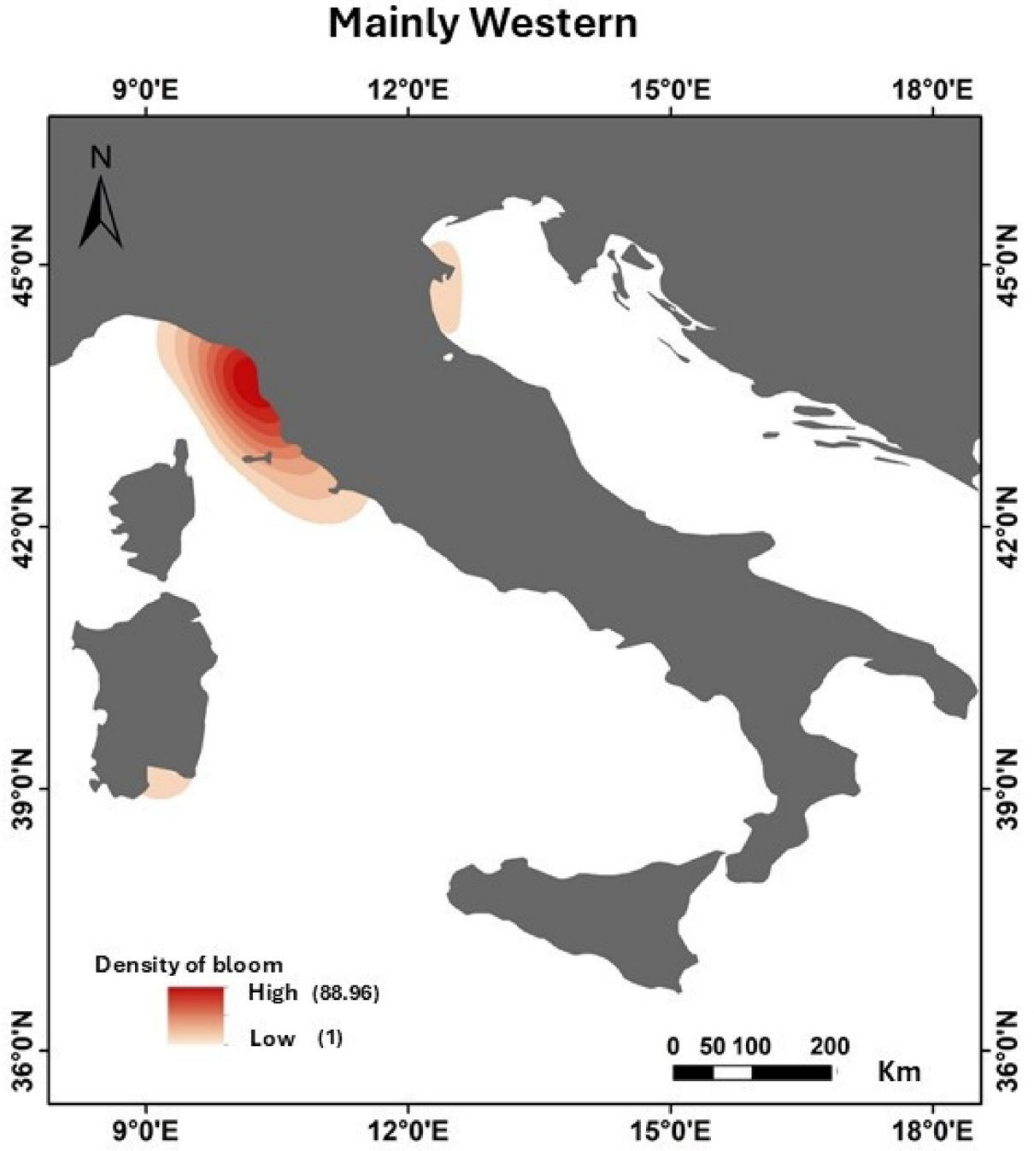
Spatial distribution of the most frequent non-indigenous species (NIS) bloom events: *Mnemiopsis leidyi*. The colour gradient represents the results of a Kernel Density analysis, indicating the density of sightings of blooms. Areas with higher densities are shaded in red, while lower densities are shown in lighter colours. Map generated with ArcMap 10.8.2 (Esri, https://www.esri.com/en-us/arcgis/products/arcmap/overview).

**Fig. 6:**
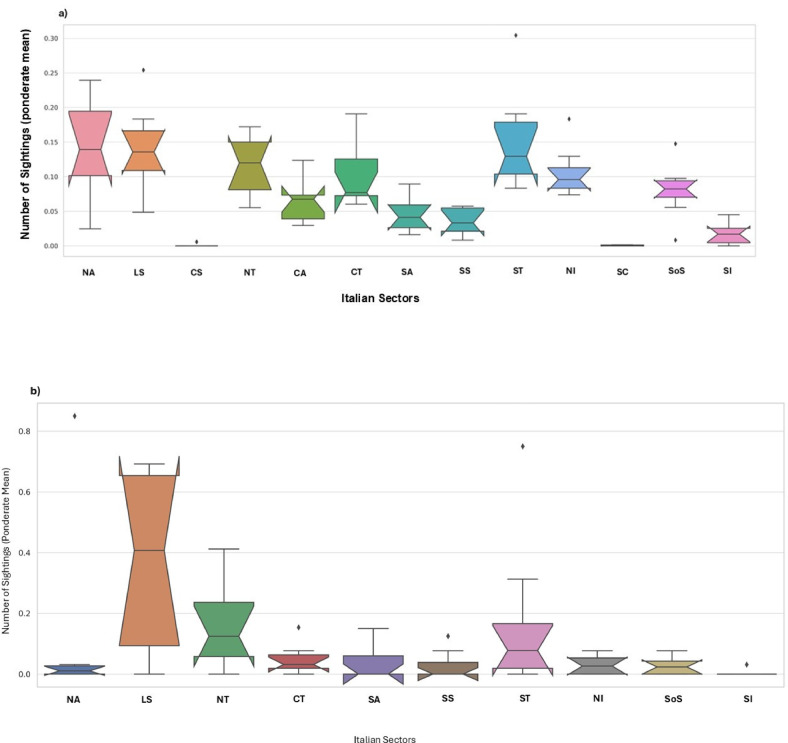
Notched box plot describing the Italian sectors of the Mediterranean in which highest number of IS (**a**) and NIS (**b**) blooms have been reported (*NA* Northern ariatic,* LS* Ligurian sea,* CS* Corsica sea,* NT*  Northern Tyrrhenian,* CA * Central Adriatic,* CT * Central Tyrrhenian,* SA * Southern Adriatic,* SS * Sardinian Sea,* ST * Southern Tyrrhenian,* NI  * Northern Ionian,* SC* Sardinian Channel,* SoS * Strait of Sicily,* SI * Southern Ionian).

The CS Program enabled the first records in Italian waters of several non-indigenous jellyfish species (NIS): *Phyllorhiza punctata* (Von Lendenfeld, 1884), *Rhizostoma luteum* (Quoy and Gaimard, 1827) (misidentified as *Catostylus tagi* (Haeckel, 1869), see^39^, *Cassiopea andromeda* (Forsskal, 1775) and *Rhopilema nomadica* (Galil, 1990)^40,41^. In addition, it facilitated sightings of rare species, such as *Drymonema dalmatinum* (Haeckel, 1880) in the northern Adriatic Sea, and the description of new species, such as *Mawia benovici*^42^ and *Aurelia relicta*^43^, newly reported in the southern Adriatic region. Each year, blooms of both indigenous (IS) and non-indigenous (NIS) taxa (see Supplementary Tab. S1 online) were reported along the eastern (n.bloom = 43%) and western (n.bloom = 57%) Italian coasts (Fig. 4, and Fig. 5) showing significant differences between sectors. Blooms of the endemic species most frequently occurred in the northern Adriatic Sea, southern Tyrrhenian Sea, Ligurian Sea and northern Tyrrhenian Sea (*P* < 0.05) and blooms of the aliens species were most frequently sighted in the Ligurian Sea and the northern and southern Tyrrhenian Sea (*P* < 0.05) (Fig. 6).

### Indigenous species (IS)

From 2009 to 2016, 6,603 blooms of IS were reported along the whole Italian coast, representing 96% of total bloom events. These were mostly due to the schyphozoans *P. noctiluca* (30%), *R. pulmo* (28%), *C. tuberculata* (12%), *Aurelia* spp. (5%); the cubozoan *C. marsupialis* (8%) and the colonial hydrozoan *V. velella* (6%). Other species, such as the hydrozoans *Olindias muelleri* (Haeckel, 1879) (1.4%), *Aequorea forskalea* (Peron and Lesueur, 1810) (1.4%), *Porpita porpita* (Linnaeus, 1758) (0.3%), the scyphozoans *Chrysaora hysoscella* (Linnaeus, 1767) (1.3%), *C. andromeda (0.2%)*,* D. dalmatinum* (0.2%), *Discomedusa lobata* (Claus, 1877) (0.03%), and the ctenophore *Leucothea multicornis* (Quoy and Gaimard, 1824) (0.08%) were frequently recorded at low abundances. Only occasional sightings were reported for the hydrozoans *Leuckartiara* spp. (Claus, 1863), *Forskalia edwardsii* (Kolliker, 1853) and *Geryonia proboscidalis* (Forsskal, 1775) and for the ctenophore *Cestus veneris* (Lesueur, 1813).

The blooms of *P. noctiluca*, *V. velella* and *C. hysoscella* were mainly sighted along the western coasts (Fig. 4). On the contrary, blooms of *Aurelia spp*,* C. marsupialis and Salpa maxima* (Forsskal, 1775) were more frequent in the eastern coasts. The blooms of *R. pulmo*,* C. tuberculata* and *A. forskalea* were widespread along all coasts. Occasional blooms were reported for *O. muelleri*, mainly in the Tyrrhenian Sea, and for *P. porpita* in the Ionian Sea and in the southern Tyrrhenian Sea.

### Non-Indigenous species (NIS)

The NIS recorded included lessepsian migrants from Suez Canal (e.g. *C. andromeda*, *Marivagia stellata* (Galil and Gershwin, 2010), *P. punctata*, and *R. nomadica*), species likely introduced through the Atlantic Sea (e.g. *M. leidyi*, *R. luteum* and *Physalia physalis* (Linnaeus, 1758)) and the newly described species *P. benovici* (Tab.S1). NIS sightings were concentrated in the Ligurian Sea (41%), followed by the northern Tyrrhenian Sea (12%) and the northern Adriatic and the southern Tyrrhenian Seas (11% each) (Tab.S1). Only two NIS accounted for 4% (286 sightings) of the total blooms observed (*M. leidyi* and *C. andromeda*) (Tab. S1). *M. leidyi* was the most recurrent blooming NIS, representing 98% of the NIS bloom sightings (Fig. 5). Blooms of *M. leidyi* were observed only along the West Italian coast, mainly in the Ligurian Sea until 2016 and on the East coast, particularly in the northern Adriatic Sea afterwards (Fig. 5). *C. andromeda* blooms were occasionally observed in the Gulf of Palermo^44^ accounting for 2% of all the NIS blooms recorded. Occasional sightings included *M. stellata*, *P. punctata* and *R. luteum* in the western Italian seas. Few individuals of *R. nomadica* were observed both in the southern Tyrrhenian Sea (in Sardinia and Sicily, respectively in 2015 and 2016) and in the northern Adriatic Sea.

*P. physalis*, the most poisonous NIS gelatinous organism usually found in the area ranging from 55° N to 40° S, was reported by citizens along the Italian coasts as single specimen stranded in the Sicilian coasts in 1999. The highest number of records of *P. physalis* was observed in the Strait of Messina (2009 and 2014), as well as in Lampedusa Island and in the southwestern Coast of Sardinia (2009 and 2021)^10,45–47^.

### Seasonality of blooms

**Fig. 7:**
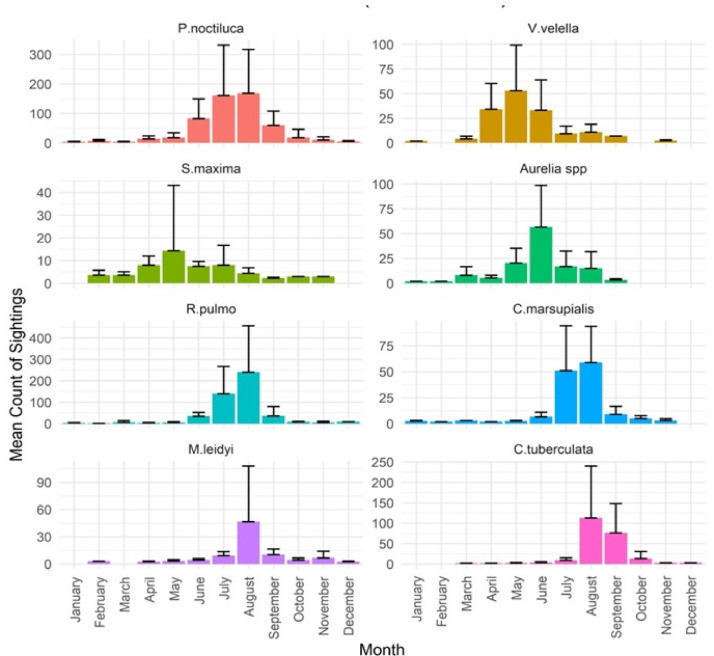
Mean Count of Sightings of the most sighted IS (*Aurelia* spp, *Caribdea marsupialis*, *Cotylorhiza tuberculata*, *Pelagia noctiluca*, *Rhizostoma pulmo*, *Salpa maxima*, *Velella velella*) and NIS (*Mnemiopsis leidyi*) with blooms reported from 2009 to 2016 and ordered by seasonal occurrence.

**Fig. 8:**
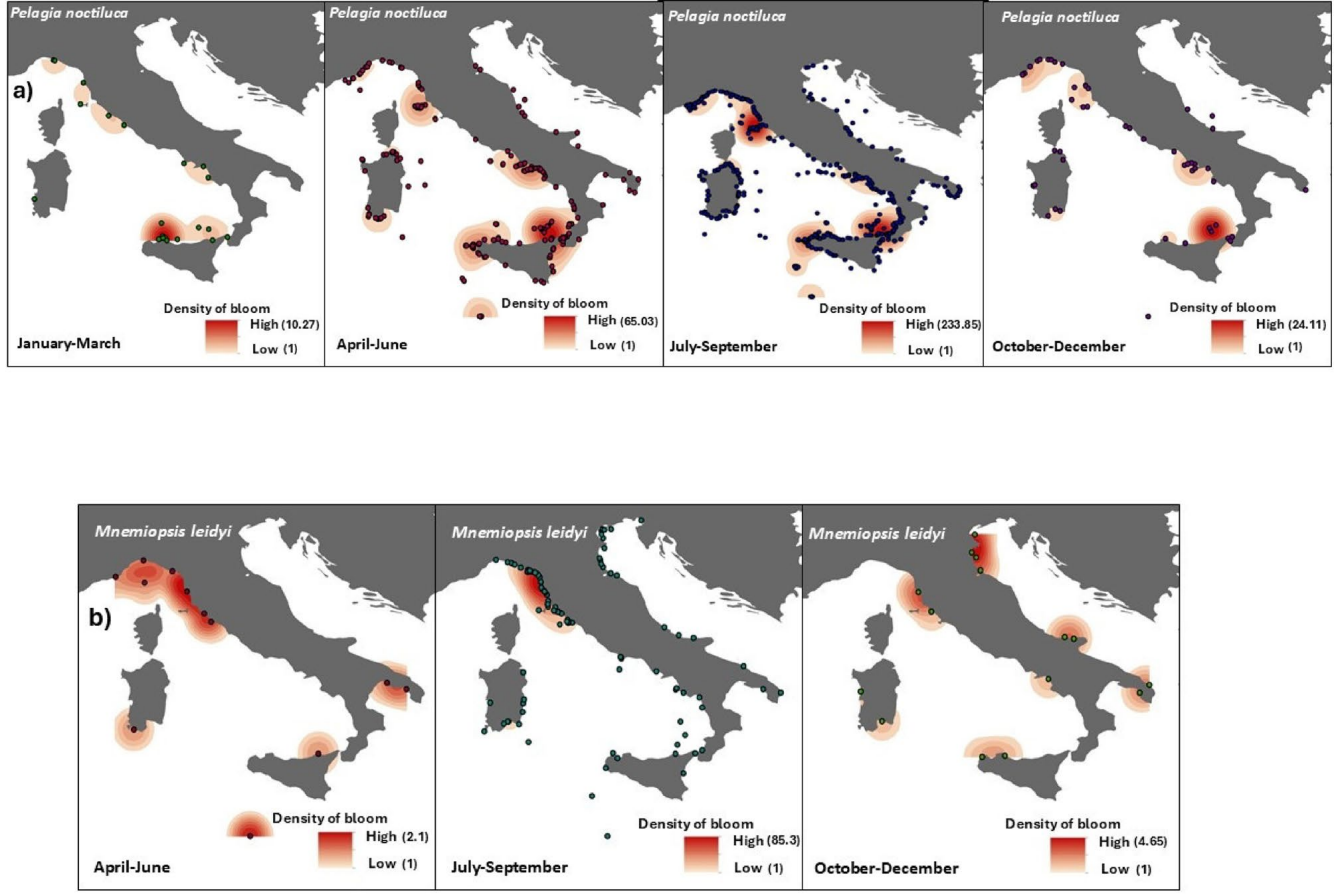
Maps showing the overall cumulative distribution of bloom events by seasons for: (**a**) the most common indigenous (*Pelagia noctiluca*), and (**b**) non-indigenous (*Mnemiopsis leidyi*) jellyfish species reported from 2009 to 2016. The colour gradient represents the results of a Kernel Density analysis, indicating the density of sightings of blooms. Areas with higher densities are shaded in red, while lower densities are shown in lighter colours. Map generated with ArcMap 10.8.2 (Esri, https://www.esri.com/en-us/arcgis/products/arcmap/overview).

Jellyfish species were present all year round along the Italian coasts, with different patterns of occurrence (Fig. 7). The mauve stinger *P. noctiluca*, the most sighted IS, was always observed, with blooms in January in the southern Tyrrhenian Sea near the coast of Sicily, and along the west coast in spring (Figs. 7 and 8), peaking from April to August. Although overall sightings during the autumn-winter period were low, blooms of *P. noctiluca* persisted in the central-southern Tyrrhenian Sea and in the Ligurian Sea; *V. velella* (Fig. 7, Fig.S5) also appeared earlier in winter, with blooms from April to June; S. *maxima* swarms were primarily observed in spring, although single specimens were recorded throughout the year. *Aurelia* spp. exhibited frequent blooms from May to August. Both *C. marsupialis* (Fig. 7, Fig. S2) and *M. leidyi* (Figs. 7 and 8) were late spring–summer blooming species, with peaks in July and August. *R. pulmo* (Fig. 7, Fig. S4) was widely distributed across all Italian marine sectors with blooms beginning in spring, increasing in frequency during summer, and persisting into winter, particularly in the northern Adriatic Sea. The remaining species did not exhibit substantial population outbreaks or notable differences in their spatial distribution.

### Overlap of jellyfish bloom hotspots

**Fig. 9:**
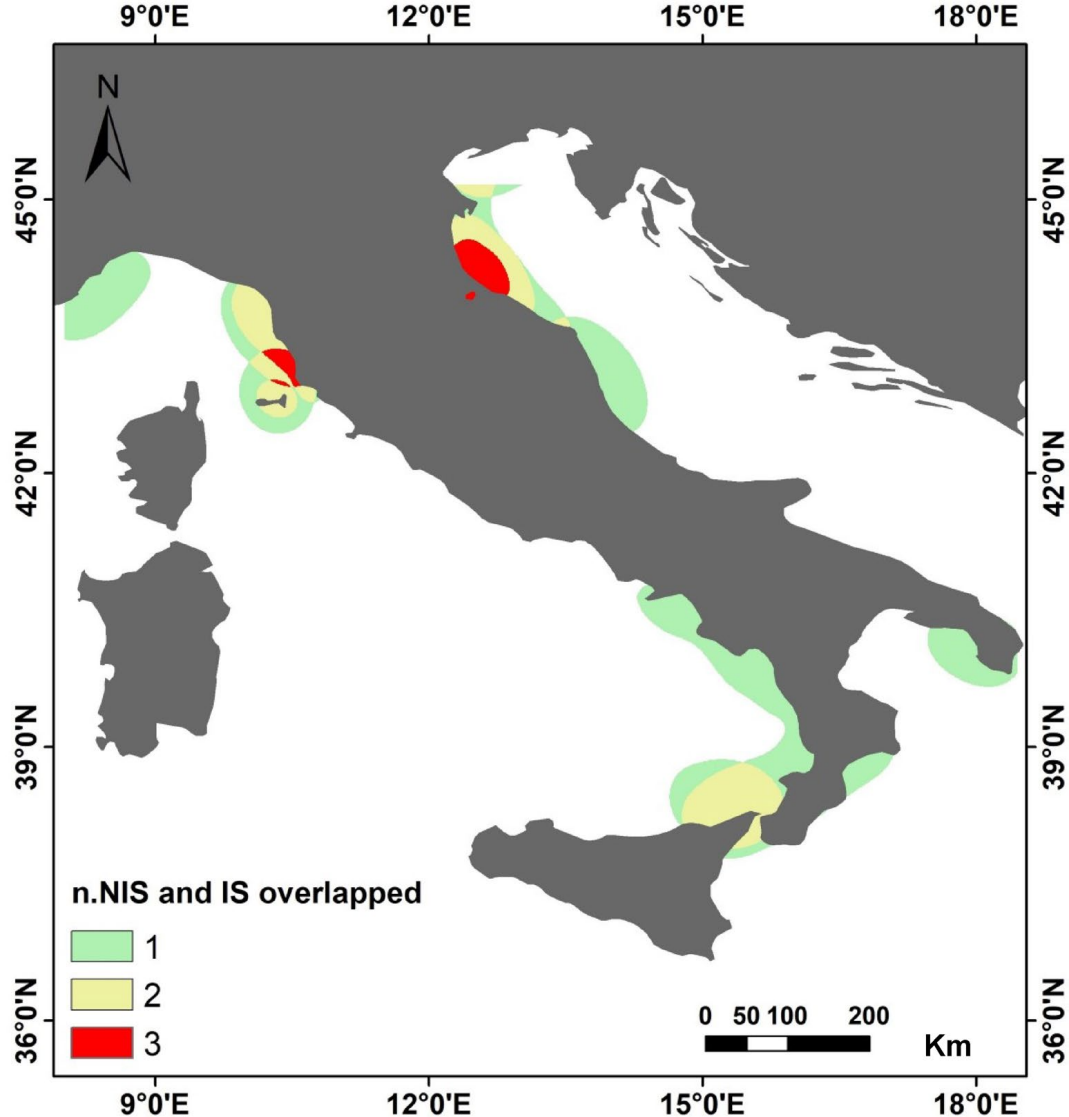
Map of Jellyfish Hotspot Overlap Index along the Italian coasts, based on kernel density estimation (60% threshold), showing areas with recurring multi-species blooms. Map generated with ArcMap 10.8.2 (Esri, https://www.esri.com/en-us/arcgis/products/arcmap/overview).

The results of the JHOI analyses revealed that the western sector of Italy, particularly the northern Tyrrhenian Sea, exhibits a significant overlap of jellyfish blooms involving three species: the indigenous species *R. pulmo* and *P. noctiluca*, and the non-indigenous species *M. leidyi*. In contrast, in the southern Tyrrhenian Sea, there is an overlap of blooms between the two indigenous species, *P. noctiluca* and *C. tuberculata*. On the eastern side of Italy, in the northern Adriatic Sea, the analysis highlighted an overlap of blooms among the indigenous species *Aurelia* spp., *C. tuberculata*, and *C. marsupialis*. These regions represent jellyfish bloom hotspots, characterized by a high likelihood of recurring jellyfish blooms that persist over the years (Fig. 9).

## Discussion

The CS data presented here, are the first consistent and spatially-extensive dataset of gelatinous zooplankton collected along the whole Italian coast. Using a citizen science-based approach, this study confirms the effectiveness of engaging the public in scientific research, filling the knowledge gaps on the presence and seasonality of gelatinous zooplankton and documenting the presence and distribution of indigenous and non-indigenous jellyfish species in the Italian seas. Citizen science data inherently depended on human presence in coastal areas. As a result, they mainly cover coastal waters leaving a significant data gap in offshore areas. Similarly, many CS observations are collected during the summer, while data from the winter months are quantitatively scarce. For this reason, in the present study, we chose to include and analyse only bloom events, considering that, although to a lesser extent, a coastal bloom is much more likely to be detected than single individuals, even during the winter season. To address these limitations, citizen science programs must be supported by environmental education initiatives aimed at the public and by the involvement of municipalities, seaside resorts, diving centres, port authorities, and the media.

Key species linked to bloom events were here identified, including the most abundant IS *P. noctiluca*, *V. velella*, *Aurelia* spp, *R. pulmo*, *C. tuberculata*, *C. marsupialis*, and the NIS *M. leidyi*. These jellyfish species were recorded the whole year round with blooms mainly reported between late-spring and the autumn.

For years, information on gelatinous zooplankton in Italian Seas have been scant and geographically restricted^48–53^, underestimating the true extent of jellyfish populations that characterise different marine regions located along 8,500 Km of Italian coastline. The interest in outbreaks of gelatinous zooplankton grew in the early 1980s, when massive blooms of *P. noctiluca* reported in many areas of the Mediterranean Sea, impacted food webs and fisheries^10^. In the late 1990s, increasing atmospheric temperatures and global warming led to warmer ocean water masses^54^ influencing hydrodynamics, nutrient distribution and oxygen levels^55^. Changing environmental conditions were then associated with increased records of jellyfish blooms^7^. The typical 12-year cycle of *P. noctiluca* blooms, documented over more than 200 years of regional observations^1^, was disrupted during the same period, with blooms of this species regularly reported ever since. This deviation supports the observed increase in Mediterranean jellyfish populations. Indeed, since the mid-1990s, massive blooms of *P. noctiluca* have been observed off Tunis, around the Balearic Islands, in the Ligurian Sea and along the coasts of Catalunia and Croatia^4,5,21,56^ also in years that were supposed to be without blooms, according to the model previously proposed by Goy et al. (1989)^1^. In recent years, *P. noctiluca* is confirmed as the most sighted stinging jellyfish in Italian waters, particularly on the west coasts, often occurring at sites closest to the upper margins of marine canyons [personal observatio^31,32,57,58^.

*V. velella* is a cosmopolitan holoplanktonic hydrozoan observed in the Mediterranean Sea since 1599^59^, mainly in the western side of the basin, as *P. noctiluca*. When blooms of this species occur, a high number of colonies swarm offshore to strand on beaches^60^. Despite *V. velella* was most frequently observed in the Ligurian Sea^61^, CS data indicated frequent stranding blooms of this species also in the Tyrrhenian Sea, especially in the south.

According to Kogovsek et al. (2010)^62^ *Aurelia* spp, *C. tuberculata* and *R. pulmo*, regularly found in the northern Adriatic Sea over the last 200 years, are the main responsible for jellyfish blooms reported in the last decades in this region. *Aurelia spp* is a cosmopolitan scyphomedusa living successfully in various habitats wherever substrates for polyp settlement are available^63^. This study confirms that *Aurelia* spp. is preferentially distributed in the northern Adriatic Sea, supporting the hypothesis of a stable population well established in this region^20,64^. In the Adriatic three different species of *Aurelia* have been reported, specifically *Aurelia coerulea,* mainly found in coastal waters, *Aurelia solida*, always recorded in offshore waters, and *Aurelia relicta*, confined to the Mljet marine lake, in the southern Adriatic^43^. However, considering that it is impossible to distinguish between *Aurelia* species only based on photographs and no physical samples were collected, sightings of *Aurelia* specimens were here referred to *Aurelia spp. R. pulmo*, primarily documented in the eastern Mediterranean Sea^20,62,89^, has been sighted mainly along the eastern Italian coast until 2012. Thereafter, its presence has increased also in the western Mediterranean, with frequency of blooms peaking in late summer and early autumn. Along the Italian coasts, *C. tuberculata* is a widespread species forming blooms with similar spatial and seasonal distribution from one year to the other, mainly in the late summer-autumn season. The Adriatic Sea hosts also the Cubozoan *C. marsupialis*, seldom reported in the literature in this region until its first bloom in 1985^65^. CS sightings indicate that *C. marsupialis* now inhabits the whole East coast of Italy and undergoes population explosions in the central Adriatic Sea during the summer-autumn seasons, immediately after the blooms of *Aurelia* spp. A shifted seasonal distribution is probably an adaptative strategy that these two species have adopted to avoid competition for food and space. *M. leidyi* is the most observed alien jellyfish species among the NIS reported in this study. Native to the Atlantic coast, *M. leidyi* has demonstrated high capability to colonize new habitats and it is now well established in the Mediterranean, having spread along the Italian, Turkish, Israel and Spanish coasts^66,67^. In Italy, *M. leidyi* was firstly recorded in the Gulf of Trieste in 2005^68^ and, during the period of our study, it has been sighted by citizens nearly annually, predominantly in the Ligurian and Tyrrhenian Seas^40^. Since 2016, citizen witnessed an increase in *M. leidyi* blooms in the northern Adriatic Sea with large swarms of this species reported along the Istrian coast from 2017 to 2018^69–73^. Other noteworthy NIS records include *C. andromeda*, which likely entered from the Suez Canal and *R. nomadica*, one of the most successful NIS jellyfish from the Levantine waters^74^. *R. luteum*, now common in Portuguese waters, and *P. physalis*, likely entered the Mediterranean Sea from the Gibraltar Strait.

The persistence of jellyfish blooms in some areas of the North Tyrrhenian and North Adriatic and in the southern Tyrrhenian Sea, suggests that these hotspots should be considered as critical zones for monitoring jellyfish bloom dynamics in Italian coastal waters. These regions are important breeding, spawning, and nursery areas for many species of pelagic and demersal fish^75,76^. Considering that predation of jellyfish on fish eggs and larvae has proven to be detrimental for fish stocks^72,77–83^, within an ecosystem management perspective jellyfish bloom in these areas should be considered as additional pressures to which fish stocks are exposed.

Indeed, among the main jellies reported along the Italian coasts, *P. noctiluca* (native) and *M. leidyi* (non-native) are capable to exert a significant predation pressure on ichtyoplankton^80,84–88^ and on crustacean plankton that fish larvae and juveniles feed upon. Other abundant species, such as *R. pulmo*, *Aurelia* spp and *C. tuberculata*, are also cited for outcompeting some commercially important planktivorous fish, for the same zooplankton prey^83^.

*P. noctiluca*, together with *C. marsupialis* and *C. hysoscella* are the most abundant dangerous jellyfish in the Italian coasts, causing frequent cases of injuries to humans due to envenomation in the hot spot areas^26,89,90^.

Given the significant impacts that jellyfish blooms might have on biodiversity, on human health and ecosystem services, the European Marine Strategy Framework Directive (MSFD) has acknowledged the importance of monitoring gelatinous zooplankton^18^. This strategy adopts an ecosystem-based management approach to achieve Good Environmental Status (GES) across European seas and thus needs to take into account for pressures associated with jellyfish blooms. Though the implementation of gelatinous zooplankton indicators in the MSFD assessment and management framework is far from being achieved, mainly due to the difficulty in integrating monitoring approaches that can provide reliable and useful jellyfish data^91^. In this perspective, the citizen science approach represents a valuable, cost-effective tool for gathering scientific information on jellyfish at broad spatial and temporal scales^31,92–96^. As demonstrated in this study, citizen science data provide a baseline to monitor jellyfish interannual fluctuations and species-specific patterns of variability, helping to identify recurrent seasonal trends and sensitive areas, i.e. jellyfish blooms hotspots, where targeted monitoring programs should be established. These programs would be valuable both for the objectives of the MSFD and for developing early warning systems to mitigate the local impacts of jellyfish blooms on tourism and on fish populations. The presence of jellyfish along the Italian coasts in the period here considered is poorly documented by standard observational programs, which would lead to a perception of lack of proof about the importance of gelatinous plankton. CS data however, substantiate the presence of gelatinous zooplankton in the Italian Seas, highlighting the need of integrating citizen science initiatives in current monitoring programs to substantially improve the assessment of gelatinous zooplankton at broad spatio-temporal scales.

## Electronic supplementary material

Below is the link to the electronic supplementary material.

Supplementary Material 1

## Data Availability

The data of this study are available on the Emodnet platform at Boero Ferdinando, Piraino Stefano, Zampardi Serena (2019). Jellyfish sightings along the Italian coastline from 2009 to 2017. Marine Data Archive. 10.14284/345.
