# Supplementary material for: Citizen science substantiates jellyfish occurrence in the Mediterranean Sea
Source: Sci Rep. 2025 Jul 1;15:21641. doi: 10.1038/s41598-025-05789-1 (PMC12214690; doi:10.1038/s41598-025-05789-1)

Citizen Science substantiates jellyfish occurrence in The Mediterranean Sea

Serena Zampardi[1(*)], Priscilla Licandro[2], Giacomo Milisenda[3], Danilo Scannella[1],

Stefano Piraino[4,5, 6]Ferdinando Boero[7,8]

[1]Institute for Environmental Protection and Research (ISPRA), BIO-CIT, 90149 Palermo Italy

[2]Stazione Zoologica Anton Dohrn, Department of Integrative Marine Ecology, Villa Comunale, 80121 Naples, Italy

[3]Stazione Zoologica Anton Dohrn, Department of Integrative Marine Ecology, Sicily Marine Centre, 90100 Palermo, Italy

[4]Università del Salento, Dipartimento di Scienze e Tecnologie Biologiche e Ambientali (DiSTeBA), 73100 Lecce, Italy

[5]National Biodiversity Future Center (NBFC), 90100 Palermo, Italy

[6]Consorzio Nazionale Interuniversitario per le Scienze del Mare (CoNISMa), 00196 Rome Italy

[7]University of Naples Federico II, Dipartimento di Biologia, 80134 Naples, Italy

[8]Consiglio Nazionale delle Ricerche, Istituto per lo Studio degli Impatti Antropici e Sostenibilità in Ambiente Marino (CNR-IAS), 16149 Genoa, Italy

*Correspondence to: serena.zampardi@isprambiente.it

Keywords

Gelatinous organisms, Non-Indigenous Species (NIS), Geographical Distribution, Bloom, Hot-Spot Areas

Supplementary Materials

Table S1. Taxa of jellyfish recorded by the CS campaign from 2009 to 2016

**Hydrozoa**	**Scyphozoa**	**Cubozoa**	**Ctenophora**	**Tunicata**
Aequorea forskalea	*Aurelia* spp	*Carybdea marsupialis*	*Cestus veneris*	*Salpa maxima*
Porpita porpita	*Aurelia relicta*		*Leucothea multicornis*	
Forskalea edwardsi	*Cassiopea andromeda*		*Mnemiopsis leidyi*	
Geyronia proboscidalis	*Rhizostoma luteum*			
Leuckartiaria spp	*Chrysaora hysoscella*			
Olindias muelleri	*Rhizostoma pulmo*			
Physalia physalis	*Cotylorhiza tuberculata*			
Velella velella	*Discomedusa lobata*			
	Drymonema dalmatinum			
	Mawa benovici			
	Pelagia noctiluca			

	Phacellophora camtschatica			
	Phyllorhiza punctata			
	Rhopilema nomadica			

Fig.S1 Maps showing the overall cumulative distribution of bloom events of *Aurelia spp* reported from 2009 to 2016. Map generated with ArcMap 10.8.2 (Esri, https://www.esri.com/en-us/arcgis/products/arcmap/overview).

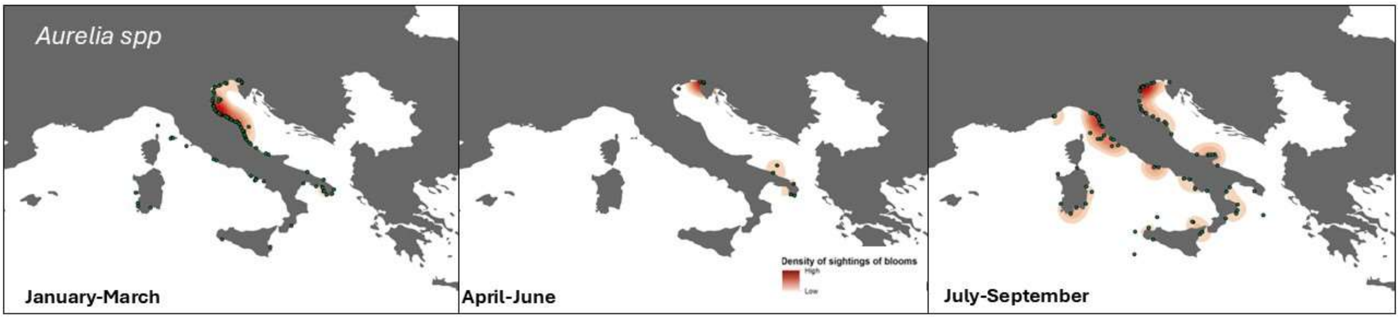

Fig.S2 Maps showing the overall cumulative distribution of bloom events of *C. marsupialis* reported from 2009 to 2016. Map generated with ArcMap 10.8.2 (Esri, https://www.esri.com/en-us/arcgis/products/arcmap/overview).

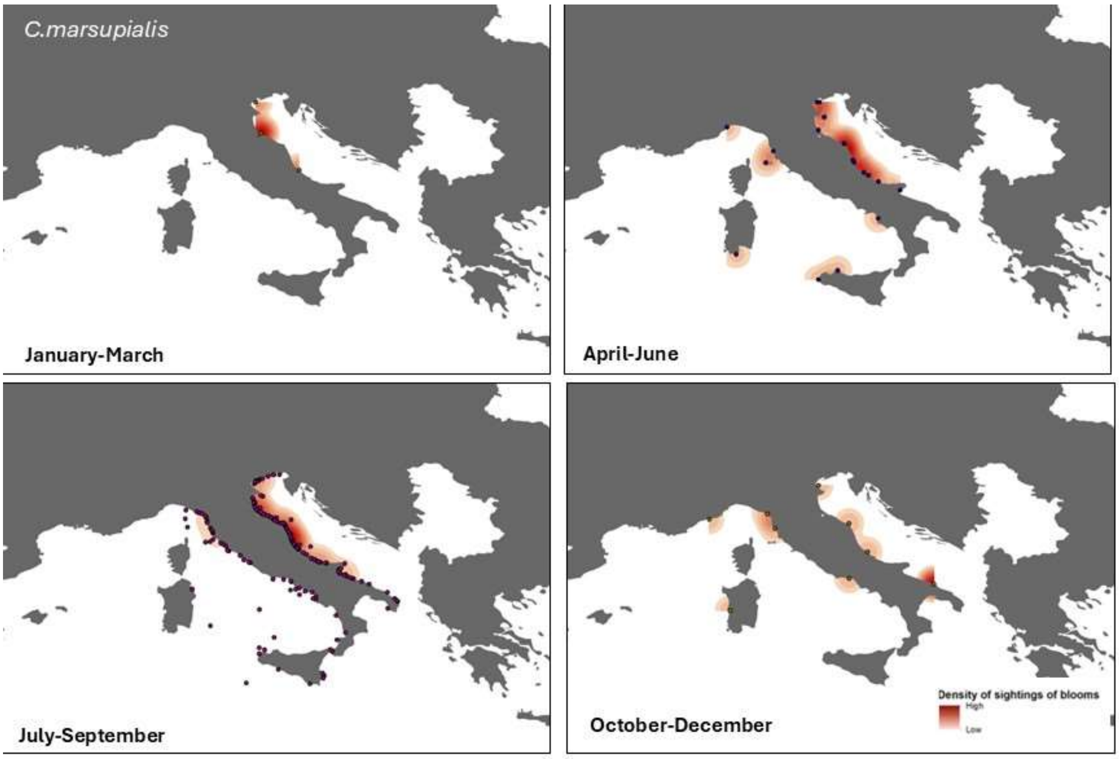

Fig.S3 Maps showing the overall cumulative distribution of bloom events of *C. tuberculata* reported from 2009 to 2016. Map generated with ArcMap 10.8.2 (Esri, https://www.esri.com/en-us/arcgis/products/arcmap/overview).

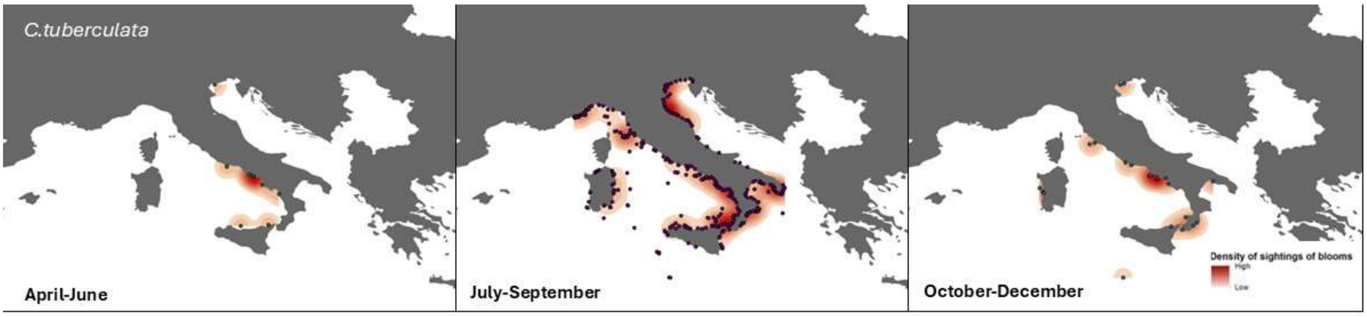

Fig.S4 Maps showing the overall cumulative distribution of bloom events of *R. pulmo* reported from 2009 to 2016. Map generated with ArcMap 10.8.2 (Esri, https://www.esri.com/en-us/arcgis/products/arcmap/overview).

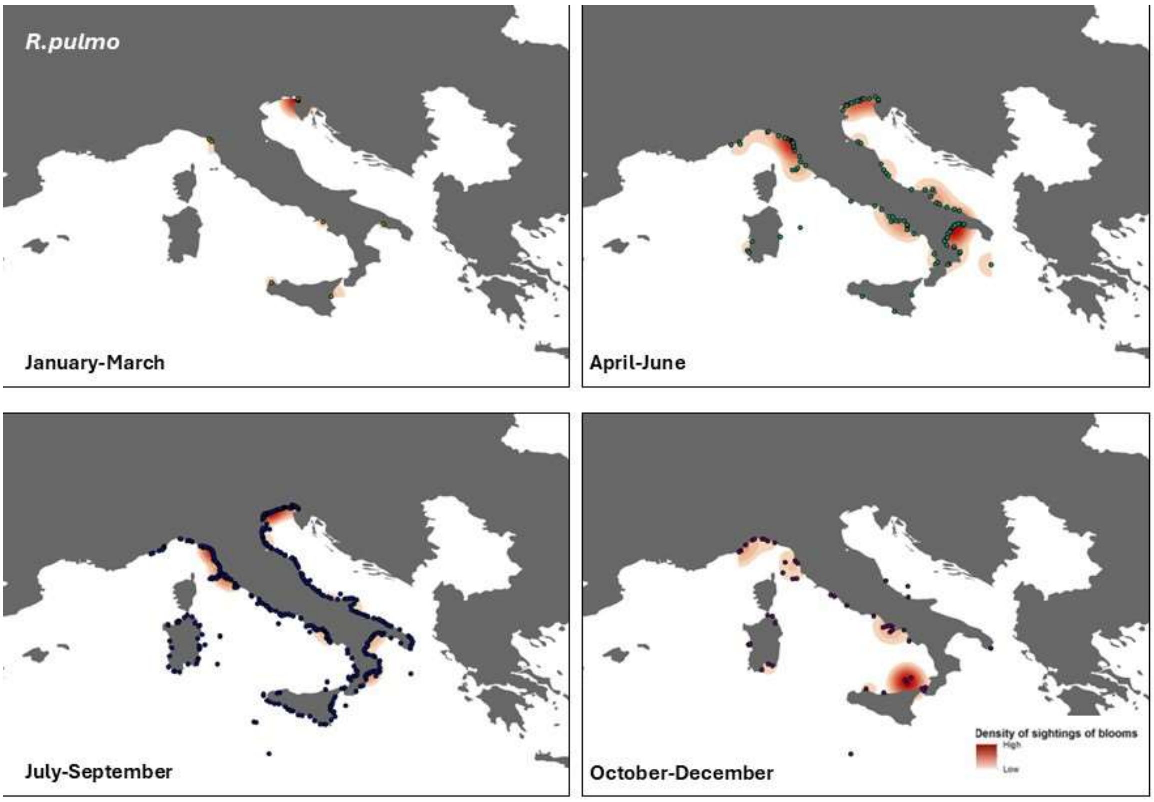

Fig.S5 Maps showing the overall cumulative distribution of bloom events of *V.velella* reported from 2009 to 2016. Map generated with ArcMap 10.8.2 (Esri, https://www.esri.com/en-us/arcgis/products/arcmap/overview).

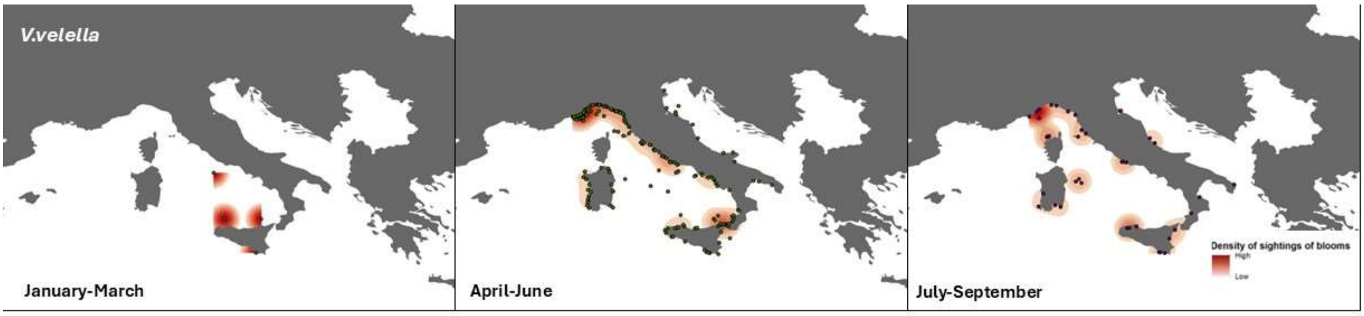

Supplement: Supplementary file 1 — Supplementary Material 1 [file 41598_2025_5789_MOESM1_ESM.pdf]
